# Poisoning by Cherry Kernels

**Published:** 1845-07

**Authors:** 


*Poisoning by Cherry Kernels.*—A daughter of a widow, æt. 5 years, ate a considerable quantity of the kernels of sweet cherries (prunus acium). Her brother (a few years older than herself), also ate some. After the lapse of a few hours, symptoms of poisoning appeared. When the author was called the next day, he found the girl so comatose, that she could not be roused by any means. The eyes were closed, pupils considerably dilated, the skin moist and hot, respiration exceedingly hurried, pulse small and quick, urine and fæces discharged involuntarily; the child very restless. A saturated solution (probably citric acid, with a carbonate of potash) was ordered internally, and cold fomentations to the head externally; after a few hours, vomiting of a greenish mass ensued, and was followed by retching, which lasted till death; the body was spasmodically contracted backwards. The illness lasted forty hours. On the *post-mortem* examination, the stomach externally was normal, and internally rather swollen and reddened; the intestines were strictured and invaginated, but there was not any inflammation. The liver, spleen, and large vessels, contained black tar-like blood. The boy, who had eaten fewer cherry kernels, became likewise ill, but recovered in the course of a month. An eruption, analogous to urticaria, came out on the fore-arms of both children; they were both perfectly well (according to the statement of the mother) before eating the cherry kernels, and no other cause for the attack could be assigned. The kernel of the prunus avium (cerasus nigra MM.) containing amygdaline, and developing prussic acid, with ethereal oil in the stomach, is a very remarkable occurrence, and one which ought to serve as a warning.—*Ibid, from Mertens of Wangroweit in der Medic. Zeit. f. Heilkunde, Prussic.*

